# The Association Between Moderate and Vigorous Physical Activity and Time to Medical Clearance to Return to Play Following Sport-Related Concussion in Youth Ice Hockey Players

**DOI:** 10.3389/fneur.2019.00588

**Published:** 2019-06-06

**Authors:** Justin T. Lishchynsky, Trevor D. Rutschmann, Clodagh M. Toomey, Luz Palacios-Derflingher, Keith O. Yeates, Carolyn A. Emery, Kathryn J. Schneider

**Affiliations:** ^1^Faculty of Kinesiology, Sport Injury Prevention Research Centre, University of Calgary, Calgary, AB, Canada; ^2^School of Allied Health, University of Limerick, Limerick, Ireland; ^3^Department of Community Health Sciences, Faculty of Medicine, University of Calgary, Calgary, AB, Canada; ^4^Cumming School of Medicine, Alberta Children's Hospital Research Institute, University of Calgary, Calgary, AB, Canada; ^5^Cumming School of Medicine, Hotchkiss Brain Institute, University of Calgary, Calgary, AB, Canada; ^6^Department of Psychology, Faculty of Arts, University of Calgary, Calgary, AB, Canada; ^7^Department of Neuroscience, Faculty of Science, University of Calgary, Calgary, AB, Canada

**Keywords:** concussion, mild traumatic brain injury, ice hockey, youth, activity

## Abstract

**Design:** Prospective cohort study.

**Background:** The recommendations regarding the optimal amount and type of rest for promoting recovery following concussion are based on expert opinion rather than evidence-based guidelines due to current a lack of high-level studies. There is an evident need for more research into the parameters of rest and activity and its effects on recovery from concussion.

**Objective:** To evaluate the association between the amount of moderate and vigorous physical activity (MVPA) during the first 3 days following concussion diagnosis and time to medical clearance (days) to return to play in youth ice hockey players.

**Methods:** Thirty youth ice hockey players (12–17 years) that were diagnosed with a concussion sustained during ice hockey were recruited to participate. The exposure was the cumulative amount of MVPA (minutes), measured using a waist-worn Actigraph accelerometer. Participants were dichotomized into high (≥148.5) and low (<148.5) activity groups based on the median of cumulative time spent in MVPA over the first 3 days following injury diagnosis.

**Results:** Participants in both the low and high activity group reported to the clinic at a median time of 4 days post-injury (low activity IQR: 3–5 days; high activity IQR: 3–7 days). The low activity group completed a median time of 110.7 min (IQR: 76.2–131.0 min) in MVPA, whereas the high activity had a median of 217.2 min (IQR 184.2–265.2 min) in MVPA. Kaplan Meier survival curves with Log-rank tests of hypothesis revealed the high activity group took significantly more time to be medically cleared to return to play (*p* = 0.041) compared to the low activity group.

**Conclusion:** The results from this study suggest that more time in MVPA early in the recovery period may result in a greater time to medical clearance to return to full participation in ice hockey. Future research, using valid measures of activity, are required to better understand the relationship between early activity and recovery following concussion in youth.

## Background

Concussion is a mild traumatic brain injury that can present with a range of symptoms that may impair an individual's ability to perform activities of daily living and result in time away from activities, such as school, work, sport, and recreational activities (1). The current guidelines for the management of acute concussion, according to the 5th Consensus Statement on Concussion in Sport, include physical and cognitive rest until acute symptoms resolve, followed by a graded program of exertion prior to medical clearance to return to play (RTP). Currently, the literature suggests that youth may take longer to recover than adults and should therefore receive the appropriate accommodations to reduce cognitive and physical load (2). Although there are conflicting reports on the efficacy of rest, expert opinion suggests an initial period of rest (24–48 h) is advised for recovery following the injury (1, 3). To date, the optimal amount and type of rest and physical activity that are most beneficial for recovery following a concussion are not well-defined (4–6).

Concussion is a result of biomechanical forces to the brain (e.g., acceleration, deceleration, rotational) that initiate a complex cascade of neurometabolic and neurochemical events that result in altered cerebral functioning (7, 8). Exercise introduced within 6 days following injury has been shown to be detrimental in animal models, whereas delayed voluntary exercise beyond 14 days appears to be beneficial (9). Thus, rest is postulated to be potentially beneficial in reducing the chances of re-injury and neuronal cell damage during the acute phase of concussion (8, 10, 11).

Complete bed rest has been described as an unrealistic and impractical prescription following concussion, and a complete absence of physical or cognitive activity is impossible (12). Participation in physical activity has been reported to have positive benefits for youth health (13). Eliminating physical activity, especially for long periods, can be expected to have a negative effect, increasing symptoms of depression and anxiety (12). Two randomized controlled trials have examined rest time on symptom scores and neurocognitive outcomes in individuals following concussion (14, 15). The first study evaluated the effects of prescribed bed rest for 6 days vs. no rest on post-traumatic complaints in participants recruited from the emergency department (14). This study found no beneficial effect on participant's reported outcomes as a result of prescribed bed rest. The second study evaluated strict rest for 5 days vs. usual care (1–2 days of rest followed by step-wise return to activity) on neurocognitive, balance and symptom assessment (15). The authors used an intention to treat analysis and found that prescribed bed rest did not improve symptoms or neurocognitive and balance outcomes. Furthermore, this study found no significant differences in the actual amount of energy expenditure and cognitive activity between groups which was measured using self-report diaries. These studies both suffer from potential measurement biases related to the use of self-reported activity diaries to monitor physical activity participation and rest rather than an objective measure of activity.

The literature provides conflicting evidence regarding the efficacy of physical and cognitive rest for reducing time to medical clearance to return to play by a physician and improving symptom scores following concussion (16–18). In regards to physical activity, Majerske et al., found that student athletes reporting moderate levels of cognitive and physical activity (e.g., school activity, jogging) during the first month after a concussion showed better neurocognitive performance and reaction time than those reporting no activity or high levels of activity (19). Further, Grool et al., demonstrated a reduced risk of persistent post-concussion symptoms at 28 days in youth (ages 5–18) who self-reported participation in early physical activity (within 7 days post-concussion) compared to those who reported no physical activity (20). Although, it should be noted that more than 2/3 of the study population reported engagement in some form of physical activity at 1 week post-injury.

To effectively evaluate the effects of physical activity on concussion recovery, reliable and valid measurement tools are imperative. Accelerometry is commonly used to measure physical activity, as it is easily administered and has been found to be valid and reliable across numerous populations (21, 22). The Actigraph accelerometer has been used in both youth and adult populations and shown to be a reliable and valid measure when compared to oxygen consumption via VO_2_ metabolic carts and other accelerometers (22–24). Whereas, self-reported activity has been shown to be an unreliable method of measuring activity, prone to a desirability bias based on the context of those observing behavior (25).

Previous literature indicates excessive rest or complete bed rest have not been shown to be beneficial for recovery from concussion; conversely, high levels of activity may slow recovery (14, 15, 19). Although previous research has attempted to evaluate the effects of activity following concussion, it is difficult to extrapolate the findings into recommendations because previous research in concussion has yet to use objective measurement devices. Therefore, studies of rest and physical activity following a concussion using improved methods for measurement are gravely needed to inform the type and amount of rest and physical activity that is most beneficial for recovery from concussion.

The primary objective of this study is to evaluate the association between moderate and vigorous physical activity (MVPA) during the first 3 days following concussion diagnosis, and time (days) to medical clearance to return to play in youth ice hockey players. Secondary objectives of this study are to examine the association between MVPA and time to: (i) return to baseline symptom scores, (ii) first day of initiation of return to play protocol, (iii) first day of unrestricted return to school.

## Materials and Methods

### Study Design

This was a sub-cohort nested within a larger cohort study that included youth ice hockey players between the who were diagnosed with concussion by a study sport medicine physician. This study was approved by the University of Calgary Conjoint Health Research Ethics Board (Ethics ID: REB15-2577). In the larger cohort study, participants reported baseline symptom scores at the beginning of the 2015–2016 ice hockey season, using the Sport Concussion Assessment Tool 3 (SCAT3) (26). The SCAT3 includes the Post-Concussion Symptom Scale (PCSS), an all-encompassing symptom scale with 22 symptoms ranging in severity from 0 (none) to 6 (severe). Thus, the corresponding PCSS severity score an individual can report is between 0 and 132. Data on demographics, concussion history, medical history and social history were collected through the Acute Sport Concussion Clinic (ASCC) at the University of Calgary Sport Medicine Center as part of the initial intake forms.

### Subjects

Male and female youth ice hockey players between the ages of 12–17 who presented to the ASCC at the University of Calgary Sport Medicine Center following a suspected concussion following an ice hockey-related concussion mechanism were approached for participation in the study. Informed written consent to participate (including parent consent and player assent if <15 years of age) was provided by all participating players. Individuals were excluded from participation in this study if they were diagnosed with an injury that was supplementary to the SRC or refused to wear the Actigraph accelerometer.

### Procedures

Players who sustained a sport-related concussion and consented to participate in this study attended an initial physician appointment, during which, they were assessed a sport medicine physician affiliated with this study. The players were required to complete standardized forms (e.g., SCAT3) and clinical tests including measures of vestibulo-ocular and cervical spine function. Concussion was diagnosed following a clinical assessment involving assessment of multiple domains that included physical signs, cognitive impairment, neuro-behavioral features, sleep disturbance and clinical symptoms which was performed by a sport medicine with clinical expertise in SRC. The physicians in the current study were blinded to the participant's physical activity levels and baseline symptom reports. Medical instructions were similar to all participants as physicians followed standard of care recommendations offered by the university clinic that advocated for participants to rest until their acute symptoms subsided followed by a gradual increase in activity using the return to play protocol (27).

### Exposure

Exposure was defined as the total amount of time (minutes) spent in MVPA in the initial 3 days (72 h) immediately following concussion diagnosis. MVPA was measured using a waist worn Actigraph wGT3X-BT accelerometer (Actigraph LLC, Pensacola, FL, USA), with raw accelerometer data categorized into activity intensities of sedentary, light, moderate, and vigorous using cut-points for adolescents previously validated by Romanzini et al. (24) Participants were asked to wear the monitor above the right anterior superior iliac spine, only taking off the monitor when bathing to prevent water damage and skin irritation. In the absence of any previously established cut-point for physical activity in a youth population recovering from concussion, the median time in moderate to vigorous activity was chosen as the time point of relevance. Due to variations in time to receive medical clearance, 3 days was chosen as the exposure window to ensure accurate collection of the participants initial physical activity after receiving medical recommendations and to ensure adherence to wearing the accelerometer.

### Outcomes

All participants completed the SCAT3 and were assessed by the physician at the initial and weekly follow-up appointments until full clearance to return to play. The primary outcome was the time from concussion to full medical clearance by a study sport medicine physician to return to full participation ice hockey. Physicians followed a standardized protocol of medical clearance to return to ice hockey, where the patient was required to be (i) asymptomatic at rest, (ii) asymptomatic with exertion, and (iii) no other reason to withhold clearance. Therefore, all participants were initially instructed to rest upon diagnosis of their SRC.

The outcome of symptom duration was defined as the number of days between the injury date and the date of return to baseline symptom scores (if available) or medical clearance by physician if no baseline was available. The outcome of number of days to the initiation of the activity portion of the return to play protocol (i.e., step 2) was recorded as the number of days from the injury date to the date the physician instructed the participant to begin the protocol. The date of return to school was self-reported by the participant to the researchers, and the number of days from the injury date was calculated.

### Statistical Analysis

The exposure of MVPA was dichotomized into high (>148.5 min) and low (≤148.5 min) time in MVPA based on the cumulative minutes spent during the first 3 days after and including the initial appointment date. The primary outcome was the number of days from the injury date to the date medical clearance to return to play (e.g., full participation in ice hockey). Secondary outcomes were the number of days from the injury date to the date of (i) return to baseline symptom scores as reported on the SCAT3, (ii) initiation of step 2 (transition from rest to light aerobic exercise) of the return to play protocol, (iii) return to full school participation. Descriptive statistics [e.g., median: interquartile range (IQR), counts (proportion)] of baseline characteristics, stratified based on low and high activity groups, were calculated. Kaplan-Meier survival analysis with log-rank tests of significance were used to evaluate the effect of high and low MVPA on time to medical clearance and the secondary outcomes. Significance was set a priori at an alpha of 0.05 for the primary outcome, with a Bonferonni correction for the secondary outcomes (0.05/3 = 0.0167). Due to the small sample size, inferential statistics assessing the effect of covariates on time to medical clearance were not possible. All statistical analyses were conducted using STATA (V.13) (28).

## Results

**Figure 1:**
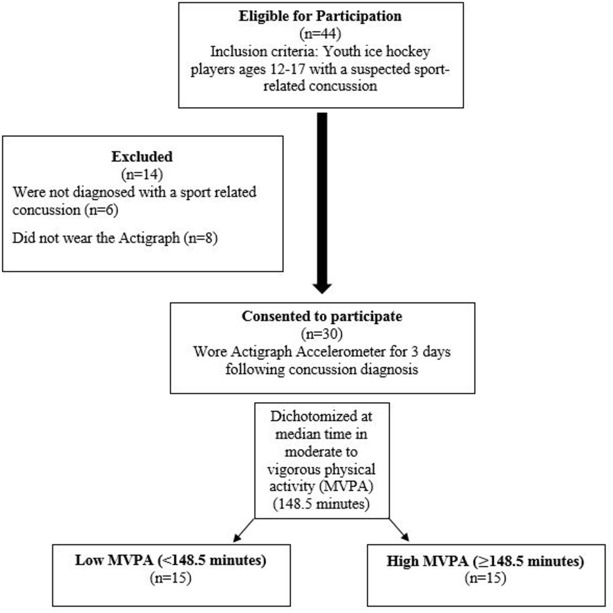
Consent flowchart.

Forty-four youth with a suspected concussion presented to the sport medicine physician during the study period (December 16, 2015–April 7, 2016). Of those, six participants were not diagnosed with a concussion at the initial appointment and were not recruited into the study. An additional 8 participants initially consented to participate but did not wear the Actigraph after enrollment in the study. These participants were excluded from all analyses. A consent flowchart detailing those who did and did not participate is depicted as Figure 1. Thirty participants were included in the current study (29), with participant demographics summarized in Table 1. Participants in both the low activity and high activity groups presented to the clinic a median of 4 days post-injury (low activity: IQR 3–5 days and high activity: IQR 3–7 days). Using the PCSS from the SCAT3, the median number of total number of symptoms that participants reported at their initial appointment was 13 out of 22 (IQR 9–20) for the low activity group and 10 (IQR 6–14) for the high activity group. The median symptom severity score for the low activity group was 31 (IQR 14–51) out of a possible 132 for the low activity group and 14 (IQR 8–29) for the high activity group. The median time in MVPA was 148.5 min (range 10.5–349.3 min) and was used to dichotomize physical activity into low and high activity. The median amount of time that the participants in the low activity spent in MVPA was 110.7 min (IQR: 76.2–131.0 min). The high activity group performed a median of 217.2 min in MVPA (IQR: 184.2–265.2 min). The median time spent in the remaining physical activity categorizations for the low and high activity groups are summarized in Table 2. The number of days from the injury date to each outcome for the low and high activity groups are presented in Table 2. Four of the total sample participants (13.3%) did not miss any days of school and returned to full school participation the day after their injury.

**Table 1:** Participant demographics.

**Characteristic**	**Low activity group**	**High activity group**
Sex	11 males, 4 females	14 males, 1 female
Age—years, median (IQR)	14 (14-15)	14 (13-15)
Height—cm, median (IQR)	166.1 (162.0–180.3)	166.2 (156.0–169.8)
Weight—kg, median (IQR)	56.6 (50.5–61.6)	53.2 (45.6–59.2)
Level of play, *n* (%)		
Elite (AAA, AA, A)	6 (40.0%)	3 (20.0%)
Non-elite (Tiers 1–7)	9 (60.0%)	12 (80.0%)
Previous history of concussion, *n* (%)		
0	9 (60.0%)	8 (53.3%)
1	4 (26.7%)	4 (26.7%)
2 or more	2 (13.3%)	3 (20.0%)
Initial total symptoms/22, median (IQR)	13 (9-20)	10 (6-14)
Initial symptom severity/132, median (IQR)	31 (14-51)	14 (8-29)
Number of days from concussion to initial appointment, median (IQR)	4 (3-5)	4 (3-7)
Median total time spent in sedentary (IQR)	3225.3 (3410.7–3400.0)	3050.0 (2908.8–3163.8)
Median total time spent in light (IQR)	269.3 (213.8–367.8)	434.7 (274.2–584.7)
Median total time spent in moderate (IQR)	72.3 (50.8–77.8)	117.0 (100.8–140.2)
Median total time spent in vigorous (IQR)	35.8 (23.3–52.0)	100.2 (80.2–127.8)
Median time in moderate to vigorous (IQR)	110.7 (76.2–131.0)	217.2 (184.2–265.2)

**Table 2:** Outcomes by activity group.

**Outcome**	**Low activity (MVPA <148.5 min)**	**High activity (MVPA ≥148.5 min)**	**Log-rank test**	
			**chi^**2**^**	***p*-value**
Number of days to medical clearance to return to play (median, IQR)	16 (11-24)	19 (14-38)	4.18	0.0409^*^
Number of days to return to baseline symptom scores (median, IQR)	15 (11-23)	19 (14-36)	4.98	0.0256
Number of days to initiation of return to play protocol (median, IQR)	11 (10-14)	12 (9-21)	1.58	0.2090
Number of days to full return to school (median, IQR)	9 (8-13)	3 (2-9)	1.47	0.2246

* *Significant difference (significance p < 0.05) in survivor function between low and high activity groups for primary outcome*.

**Figure 2:**
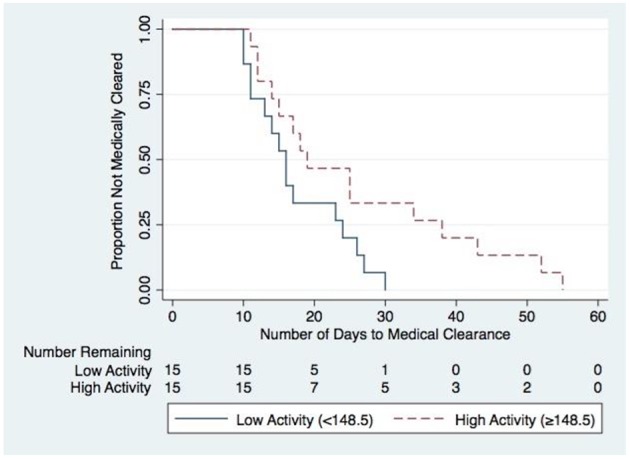
Kaplan Meier survival curves for high and low MVPA.

Survival analysis using log-rank tests were used to compare the low and high activity groups for the primary and secondary outcomes. The results of these tests are presented in Table 2. The high activity group took significantly more time to be medically cleared to return to play (chi^2^ = 4.18, *p* = 0.041) compared to the low activity group (Figure 2). Following Bonferroni correction for the remaining three outcome measures (0.05/3 = 0.0167), there were no significant differences identified in any of the secondary measures between low and high activity groups.

## Discussion

This is one of the first studies to objectively assess the association of high and low different levels of physical activity using accelerometry during recovery with time to medical clearance to return to play. All participants in the current study performed at least some MVPA in the first 3 days after diagnosis, despite receiving instructions from their treating physician to rest following the initial appointment. Youth athletes in the current study who performed greater amounts of MVPA in the first 3 days following concussion diagnosis, which was within 7 days post-injury for most, experienced significantly greater time to receive medical clearance to return to sport. A study by Grool and colleagues demonstrated that youth who self-reported early physical activity participation, which was primarily in the form of light aerobic exercise, had reduced risk of developing persistent symptoms compared to those who did not engage in any physical activity (20). Complete rest following concussion is unreasonable as youth participants are unlikely to comply (20). Instead of completely eliminating initial activity following a concussion, efforts should perhaps be made to limit the amount of higher intensity activities.

Our results suggest that more time spent in MVPA during the first 3 days following initial assessment and concussion diagnosis is associated with a greater time to medical clearance to return to play (i.e., full participation in ice hockey). These findings are different than the results of Howell et al., who reported that in adolescents, higher levels of physical activity and lower initial symptoms after concussion are associated with a shorter duration of symptoms (27). However, this may be due higher physical activity being associated with a less severe injury, as these individuals may be less symptomatic upon resuming activity and thus become asymptomatic sooner. Howell et al., also used a self-reported activity questionnaire, recording participants' activity levels during the entire duration of their recovery, which may be a source of reporting bias leading to different results than this study (30).

All participants in this study reached each outcome in a logical order by returning to full school participation first, followed by starting the return to play protocol, then returning to baseline symptom scores before being medically cleared. The low activity group obtained medical clearance for full participation in ice hockey in fewer days than the high activity group, however, the high activity group returned to school sooner.

## Limitations

Due to the sample size, we were unable to evaluate the effect of additional covariates on time to medical clearance. Collectively, individuals in the low activity group appeared to have higher median PCSS symptom severity scores on presentation, although no statistical tests were performed. Previous research has suggested that total symptom severity score may be a possible confounder on time to recovery, as greater symptom burden would likely reduce initial physical activity levels and may lead to a longer recovery time (31, 32). Even despite higher PCSS score upon presentation, individuals in the low activity group achieved medical clearance to return to sport sooner than their high activity counterparts. Future studies evaluating time to medical clearance should include rigorous evaluation of these symptom-related covariates.

The difference between the Kaplan-Meier curves in the number of days to full return to school was not statistically significant. The point estimates for the median time of full return to school for the high activity group was smaller than for the low activity group. The high activity group returning to school earlier may have provoked and prolonged their symptoms, resulting in a longer time to return to baseline symptoms and time to obtain medical clearance. More research evaluating both physical and mental activity across the entire duration of recovery is needed.

Personality traits, such as risk-taking social behaviors may have also contributed to a stunted recovery for those in the high activity group. Future research should concurrently monitor social behavior and physical activity to determine its influence on recovery.

In the absence of any previous well-defined parameters for rest or physical activity for youth following concussion, the median of 148.5 min over the course of 3 days of MVPA was selected as the cut-point for high and low physical activity and has no known clinical significance. Three days was chosen in order to ensure adherence by all participants to wearing the accelerometer. Future studies with means of accurately tracking activity for an extended period of time, all the while maintaining compliance to wearing the device, are needed.

Examining the relationship between sedentary time and recovery from concussion is also important. Future research with larger sample sizes are needed to conjointly evaluate the amount of rest while taking into account MVPA is needed.

The participants in this study collectively reported to the clinic at a median of 4 days (IQR: 3–6 days) after their concussion and therefore the type and amount of physical activity that participants engaged in immediately after their concussion remains unknown. Thus, another potential confounder is the time to presentation to the sports medicine physician with a suspected concussion. Additionally, it is unknown what activity participants were doing immediately after the injury and how this would affect recovery.

Participants self-reported the date they first returned to school. The date of return to school may have been influenced by the timing of the date of the injury (i.e., if the injury occurred with an ensuing school break, the first date of return to school occurred after the break). This could have led to an over estimation of the amount of time to return to school, although we would expect this to be similar between both groups.

A Hawthorne effect may have occurred for both physicians and participants. Participants wore the accelerometer on the waist and were aware that their activity was being observed and measured. Knowing that they were being studied, participants may have then changed their behavior and activity may appear more compliant with the physician's instruction to rest in the initial time period following concussion and then gradually complete the return to sport protocol. This could lead to an underestimation of the true activity that may have otherwise been performed by participants if they were not having activity tracked. However, this effect is not expected to differ between study groups. Physicians may have also acted more conservatively in granting their return to play decision if they felt their decision was being monitored by the researchers in this study. This may have led to an overestimation of the actual amount of time, determined by the physician, to become ready for re-participation in sport. Again, this would not be expected to be different between the low and high activity groups.

The treating physicians in the current study followed their patients from injury diagnosis to medical clearance and followed standardized return to play protocols. This was done to reduce measurement error through inter-rater differences, which would have been present if multiple physicians were involved in the treatment of a single subject. Multiple physicians were allotted to the treatment of participants in this study. Therefore, although standardized procedures were in place, measurement error could have occurred if there were systematic differences between physicians in their clinical judgment and their decisions to grant medical clearance. Future studies could address this potential limitation by operationalizing the instructions given to patients and further standardizing the criteria to obtain medical clearance.

## Conclusion

Youth ice hockey players that participated in MVPA for more than 49.5 min per day, in the first 3 days after their initial assessment took significantly longer to receive medical clearance to return to play than players participating in <49.5 min of MVPA per day. Currently, the optimal amount and type of rest and physical activity that are most beneficial for recovery following a concussion are not well-defined (4–6). Complete rest following concussion diagnosis is unreasonable as individuals, especially youth, are unlikely to comply. Previous research has shown that early initiation of light intensity physical activity may facilitate recovery (20). Whereas, our results suggest that more time spent in MVPA during the first 3 days following concussion diagnosis is associated with a greater time to medical clearance to return to play. Thus, recommendations to limit the amount of time in MVPA initially for adolescent athletes may facilitate recovery following concussion.

## Ethics Statement

This study was approved by the University of Calgary Conjoint Health Research Ethics Board (Ethics ID: REB15-2577). Informed written consent to participate (including parent consent and player assent if <15 years of age) was provided by all participating players.

## Author Contributions

JL and TR provided substantial contributions to the identifying the research question, acquisition of data and analysis, and development of the manuscript. The remaining authors critically revised all aspects of the manuscript and provided intellectual contributions. The corresponding author oversaw and contributed to all aspects of this project. All authors provided approval for publication of the content.

### Conflict of Interest Statement

The authors declare that the research was conducted in the absence of any commercial or financial relationships that could be construed as a potential conflict of interest. The handling editor declared a past co-authorship with several of the authors [KY, CE, and KS].
